# Functional poly(ether-ketone-ketone) composite scaffold with enhanced cell-material interaction, anti-inflammatory and osteogenesis for facilitating osteointegration and bone regeneration

**DOI:** 10.1016/j.mtbio.2025.101533

**Published:** 2025-01-29

**Authors:** Qianwen Yang, Anbei Chen, Xin Zhang, Zhaoying Wu, Chao Zhang

**Affiliations:** School of Biomedical Engineering, Shenzhen Campus, Sun Yat-Sen University, Shenzhen, Guangdong, 518107, China

**Keywords:** Poly(ether-ketone-ketone), Cell adhesion, Osteoblastic differentiation, Anti-inflammatory, Osteointegration

## Abstract

Bone defects resulting from trauma or disease remain a significant challenge in clinical practice, often requiring prolonged treatment. Poly(ether-ketone-ketone) (PEKK) is a commonly used implant material due to its excellent biocompatibility and mechanical properties, which are similar to those of bone. However, its biological inertness leads to poor anti-inflammatory and osteointegration properties, significantly hindering the bone repair process. In this study, a cryogel filled - PEKK/bioglass (BG) composite scaffold (SPBC) was prepared *via* 3D printing to provide immunomodulatory and bone integration performance. Compared with untreated PEKK, SPBC exhibited significant enhancements in surface properties, including higher hydrophilicity and roughness. Additionally, SPBC enhanced the adsorption of fibronectin and vitronectin on the scaffold surface and regulated the maturation of cytoskeleton and adhesion plaques by increasing the phosphorylation level of FAK at Y397, thereby promoting cell adhesion and spreading. Due to the release of bioactive ions, SPBC can significantly promote the polarization of RAW264.7 cells towards M2 and the secretion of anti-inflammatory cytokines, while also enhancing the proliferation and differentiation of rat mesenchymal stem cells (rMSCs) *in vitro*. Furthermore, the *in vivo* results confirmed the enhanced anti-inflammatory properties and the integration of SPBC with the host tissue. In summary, after surface modification and cryogel filling, SPBC demonstrated excellent anti-inflammatory and bone integration abilities, presenting potential for clinical application as an orthopedic implant scaffold.

## Graphical abstract

**Figure:**
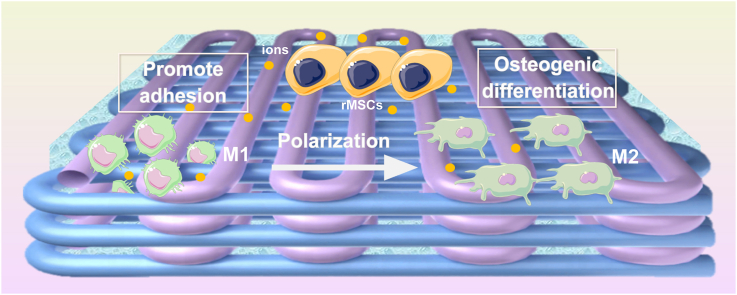

## 1 Introduction

Poly(ether-ketone-ketone) (PEKK) is a polymeric material known for its easy processing and elastic modulus comparable to that of human bone, offering a key advantage of reduced stress shielding in comparison to metal implants [1,2]. In addition to these characteristics, PEKK exhibits exceptional heat resistance, corrosion resistance, radiolucency, and MRI compatibility, making it an attractive material for bone implants in the biomedical field [3]. For implants, achieving optimal osteointegration with the host bone and providing excellent immunomodulatory properties are essential prerequisites for both early fixation and long-term stabilization in bone [4]. However, the limited interaction between PEKK and cells can lead to inflammatory responses, poor osteointegration, and an elevated risk of implant failure. Hence, enhancing the biological activity of PEKK-based implants remains a significant challenge in this field [5].

The increasing understanding of cell-material interactions has led to the recognition that scaffolds play a pivotal role in providing cells with crucial cues for guiding their behavior and regulating the local tissue microenvironment [6]. The initial poor osseointegration is attributed to the adverse interface between PEKK and host bone tissue, hindering osteoblast-related cells adhesion, proliferation, and differentiation on the implant surface [7]. To address this issue, various approaches (acid corrosion, plasma processing, layered assembly, physical adsorption) have been developed to enhance the biological activity of PEKK [[8], [9], [10]]. Notably, the main chain of PEKK contains a similar light initiator, diphenyl ketone, which may generate free radicals under UV irradiation-induced graft polymerization [11]. Gelatin methacryloyl (GelMA) is a highly attractive material due to its favorable biocompatibility, low immunogenicity and photo-crosslinkable structure. Its molecular chain on Arg-Gly-Asp (RGD) sequence promotes the biological interaction between cells and scaffolds [12]. Additionally, the freeze-cast GelMA cryogel with a 3D porous structure is conducive to the diffusion of nutrients, oxygen and metabolic wastes, while simultaneously exhibiting soft tissue-like elasticity and mechanical strength [13]. So, it is reasonable to explore the potential of combining GelMA cryogel with PEKK for improving the performance of PEKK-based implants.

The osseointegration is influenced not only by the cell resident and adhesion, but also by the local immune microenvironment [14]. Macrophages, as pivotal cells within the immune system, play a crucial role in the biological response to implants [15]. When the scaffold is implanted, the activated M1 macrophages will secrete amounts of pro-inflammatory cytokines (TNF-α, IL-1β, IL-6, NO) to regulate the growth, activation, differentiation, and homing of immune cells to the site of infection, ultimately controlling and eradicating intracellular pathogens. However, the persistent activation of M1 macrophages may induce the chronic inflammation and form a thick fibrous encapsulation layer around the implant [16]. This layer not only physically isolates the implant from the surrounding bone tissue but also impedes the achievement of stable osseointegration. Therefore, it is important to timely polarize M1 macrophages towards M2 phenotype. They can secrete anti-inflammatory factors, which serve to dampen the excessive inflammatory response and create a favorable microenvironment for tissue regeneration. The anti-inflammatory effect of implants is affected by their morphology, composition, or biological function ions [17]. Bioglass (BG) is an inorganic amorphous material with excellent osteoconductivity and degradability [18]. The incorporation of trace elements like copper, silicon and strontium has been shown to reduce inflammatory responses and promote tissue repair [19,20]. Different ions have different anti-inflammatory mechanisms [21], such as the HIF signaling pathway [22], Wnt5A/Ca^2+^ pathway [23] and TLR pathway [24]. Therefore, it can be surmised that the incorporation of BG into PEKK may confer immunomodulatory function.

To design a PEKK-based bioactive composite with anti-inflammatory and cell adhesion properties, PEKK/BG composite was prepared and the pores of the sulfonated scaffolds were filled with GelMA cryogel (SPBC). The surface characteristics and ions release of SPBC were tested. Furthermore, the effects of SPBC on the adhesion and osteogenic differentiation of rat mesenchymal stem cells (rMSCs), and polarization of RAW264.7 cells were evaluated *in vitro*. Finally, the *in vivo* osteogenesis and osseointegration effects of SPBC were evaluated using rat femur repair experiments, and the underlying molecular mechanism for these effects of SPBC was further unraveled.

## 2 Materials and methods

1) Preparation of bioglass and composite scaffolds

BG was prepared by sol-gel method and post impregnation method. Briefly, 25 mL anhydrous ethanol and 3 mL tetraethyl orthosilicate were added into 8.2 mL ethanol, 12.4 mL water, and 4.5 mL ammonia. The stirring speed was decreased from 900 rpm to 300 rpm for 30 min. Subsequently, 0.64 g Ca(NO_3_)_2_·4H_2_O and 0.57 g Sr(NO_3_)_2_ were added to the solution and stirred for 1.5 h. The resulting mixture was centrifuged for 5 min, and the supernatant was poured to obtain a white powder product, followed by cleaning with ethanol and deionized water, respectively. The powder was dried in an oven at 60 °C overnight and subsequently sintered at 600 °C for 2 h to obtain SrBG. After soaking 1 g SrBG in 0.2 M Ce(NO_3_)_3_·6H_2_O ethanol solution for 24 h, the cleaning and drying processes were the same as above. Finally, BG was obtained by sintering it at 600 °C for 2 h.

PEKK and BG were mixed into wires using a micro twin-screw extruder, and 3D printed PEKK/BG composite scaffolds were prepared by fused deposition modeling technique. The nozzle temperature was 360 °C, the printing speed was 20 mm/s, the filament width was 0.35 mm, and the filament spacing was 1.1 mm. The scaffolds were sequentially cleaned with alcohol and ultrapure water, and dried in a vacuum drying oven at 60 °C. As a control group, PEKK scaffolds were shortly named P. The scaffolds were sulphonated with concentrated sulfuric acid for 1 min, immersed in deionized water for 5 min, and hydrothermally treated in an autoclave at 100 °C for 4 h. Then the sulfonated PEKK scaffolds (shortly named SP) and the sulfonated PEKK/BG composite scaffolds (shortly named SPB) were prepared.

SPB was first immersed in a solution of 10 % GelMA, exposed to UV for 90 min, and then immersed in 5 % GelMA solution with 0.25 % LAP. The scaffolds were frozen at −20 °C for 2 h, and irradiated with 405 nm UV on ice for 1min. After melting process at room temperature, the sample was sequentially immersed in 75 % alcohol and PBS for cleaning. The sulfonated PEKK/BG composite scaffolds covalently grafted with cryogel were labeled SPBC, and the synthetic scheme is provided in Fig. S1a.

2) Characterizations

The morphology and element composition of BG and scaffolds were detected using scanning electron microscopy (SEM, Quanta 400F, FEI/OXFORD) equipped with energy-dispersive spectroscopy (EDS). The interface bonding was tested using electron paramagnetic resonance (EPR, Bruker EMX PLUS) and Fourier transform infrared spectrometry (FTIR).Thermal stability analysis (TG) was performed using a thermal weight loss analyzer under nitrogen atmosphere. The heating rate was 10 °C/min, and the heating range was from room temperature to 800 °C. The average roughness values (Sa) of the scaffolds were determined using a laser microscopy (Shape measurement laser microscope, VK-150K, Keyence). The contact angle of water was measured with a contact angle goniometer. The mechanical properties of scaffolds were evaluated by a universal testing machine (AGS-X-50N, SHIMADZU) with a constant strain rate of 0.5 mm/min.

To evaluate the mineralization of scaffolds *in vitro*, the various samples were immersed into simulated body fluid (SBF) for 14 days. The ion release concentrations of Si, Ca, Sr and Ce in Tris-HCl (pH = 7.4) were measured using an inductively coupled plasma atomic emission spectrometer (iCAP Qc, Thermo Fisher Scientific) after immersing the samples at 1, 2, 3, 7, 11 and 15 days.

3) Protein adsorption

The protein adsorption capacity of scaffolds was measured both *in vivo* and *in vitro*. Fluorescein isothiocyanate (FITC)-labeled bovine serum albumin (BSA) was used *in vitro*. The scaffolds (n = 3) were placed in 10 μg/mL FITC-BSA -PBS solution at 37 °C for 4 h, followed by a gentle wash with PBS to remove any residual protein. The concentration of adsorbed protein was measured using a microplate reader at 525 nm, and a standard curve of FITC-BSA was established.

For protein adsorption *in vivo,* the scaffolds were implanted in the distal femur of rats for 24 h and then rinsed with PBS to remove the unattached protein. The scaffold was immersed in RIPA (containing 20 % proteinase inhibitor) for 1 h on ice. After that, the solution was centrifuged at 12000 rpm at 4 °C for 20 min, and the supernatant was collected and the total protein concentration was measured with a BCA kit. Western blotting was performed to detect the expression of fibronectin and vitronectin adhering to the scaffolds.

4) Cell culture

RAW264.7 cells were seeded onto 96-well plates at a density of 5.0 × 10^3^ cells per well and then cultured with four different scaffolds to evaluate cell proliferation using the Cell Counting Kit-8 (CCK-8) assay. In the cell residence assay, 1 mL cell suspension contained 5.0 × 10^5^ cells. For immunofluorescence and flow cytometry, RAW264.7 cells were seeded onto the scaffolds at 5.0 × 10^5^ cells per well in 24-well plates. After 3 days, cells were labeled with PE anti-mouse CD86 antibody and FITC anti-mouse CD206 antibody (Biolegend, USA). The proportion of M2 (CD206^+^) macrophages was determined *via* flow cytometry. Supernatants were harvested and cytokine concentrations of TNF-α and IL-10 were quantified by ELISA (Jianglai, China) at 1, 3 and 5 days.

5.0 × 10^5^ rMSCs were seeded on the surface of scaffolds for 2 h, 12 h and 24 h and the cytoskeleton was stained with phalloidin to characterize the adhesion of cells. CCK-8 was used to test the cytotoxicity and proliferation of rMSCs on scaffolds. Immunofluorescence staining of vinculin was distinguished. Briefly, 2.0 × 10^3^ rMSCs were co-cultured with scaffold for 24 h. Cells were stained with anti-vinculin antibody (Abcam, HK) and Alexa Fluor 647-labeled goat polyclonal secondary antibody to rabbit IgG-H&L (Abcam, HK). The samples were observed with a confocal laser scanning microscope (CLSM, A1, Nikon, Japan). ALP and ARS were tested to evaluate osteogenic differentiation after incubation for 7 and 14 days. The expression of osteogenesis-related genes (encoding ALP, Runx2, Collagen I and OCN) was detected by qPCR. The fold change was calculated using the 2^−ΔΔCT^ method.

5) Western blot analysis

5.0 × 10^5^ rMSCs were co-cultured with scaffolds for 1 day and then immersed in RIPA (containing 20 % proteinase inhibitor) for 20 min on ice to obtain protein solution. Protein samples were mixed with SDS-PAGE loading buffer at 4:1 ratio and heated at 99 °C for 8 min. The protein lysates were subject to fractionation through 8 % SDS-PAGE and transferred to a PVDF membrane (Merck Millipore, USA). After blocking with 5 % BSA in TBST (100 mM Tris–HCl, pH 7.4, 150 mM NaCl, with 0.1 % Tween-20), the membrane was incubated with primary antibodies overnight at 4 °C. Membranes were then washed three times with TBST and incubated for 1 h at room temperature with the appropriate secondary antibody. After washing with TBST for three times, immunoreactivity was detected using the ECL kit (Monad, USA). The relative densities of the protein bands were analyzed with Alpha-FluorChemQ imaging system. The primary and secondary antibodies used were as follows: anti-Fibronectin (ZenBio, China), anti-Vitronectin (ZenBio, China), anti-FAK (Abcam, UK), anti-p-FAK (Abcam, UK), anti-β-actin (Proteintech, USA), goat anti-mouse immunoglobulin G (IgG) H&L [horseradish peroxidase (HRP)] (ZenBio, China) and goat anti-rabbit IgG H&L (HRP) (ZenBio, China).

6) Distal femur defect model in rats

Forty-five male SD rats (300 g, 8–12 weeks) were anesthetized with Zoletil 5 (30 mg/kg) and Xylazine hydrochloride (5 mg/kg) *via* intraperitoneal injection. All animal experiment were approved by the Institutional Animal Care and Use Committee (IACUC), Sun Yat-Sen University Animal Ethics Committee of Shenzhen Medical Device Testing Center (SYXK (粤) 2021-0112) and followed the institutional guidelines. The three scaffolds (Φ3 × 4 mm) were implanted in the distal femur of rats. After euthanizing the rats at days 3, 14, 28, 56 and 84 post surgery, samples were collected and fixed in 4 % paraformaldehyde. In the early stage (day 3 and 14), the hard tissue sections (10 mm) were analyzed by immunofluorescence (IF) to distinguish between M1 and M2 phenotype macrophages, employing the anti-CD86 (Santa Cruz, USA) and anti-CD206 antibodies (Proteintech, USA) respectively. ImageJ software was employed for the quantitative analysis of the number and proportion of CD86^+^ cells (M1) and CD206^+^ cells (M2). Samples at 4, 8, and 12 weeks were subjected to high-resolution micro-CT analysis (Scanco Medical, Switzerland). H&E staining and Masson's trichrome staining were conducted to assess the new bone tissue. The collagen volume fraction and bone-implant contact were calculated from the results obtained from the Masson's trichrome staining pictures at 12 weeks.

7) Statistical analysis

All the results were shown as mean ± SD in this study. One-way ANOVA analysis was conducted when there were three or more conditions. Significant differences are presented as follows: ∗*p* < 0.05, ∗∗*p* < 0.01, ∗∗∗*p* < 0.001.

## 3 Results

### 3.1 Characterization of BG and scaffolds

**Fig. 1:**
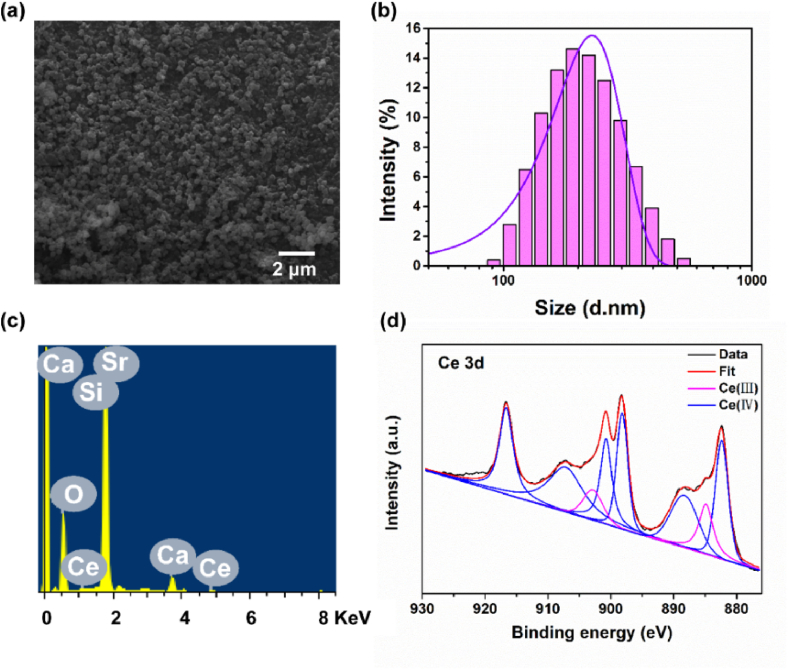
(a) SEM image, (b) diameter, (c) EDS analysis and (d) XPS spectra of bioglass (BG).

BG exhibited a spherical morphology with an average diameter of approximately 222.91 nm (Fig. 1a and b). The elements silicon (Si), calcium (Ca), strontium (Sr), and cerium (Ce) were found to be uniformly distributed throughout the BG particles, with a molar ratio of 76.0:13.8:7.9:2.3 for these four elements (Fig. 1c). To further investigate the oxidation states of Ce within the BG composition, XPS analysis was conducted (Fig. 1d). The results revealed the presence of two distinct valence states of Ce, namely Ce (III) and Ce (IV), with the former accounting for approximately 14.4 % of the total Ce content and the latter representing approximately 85.6 %.

**Fig. 2:**
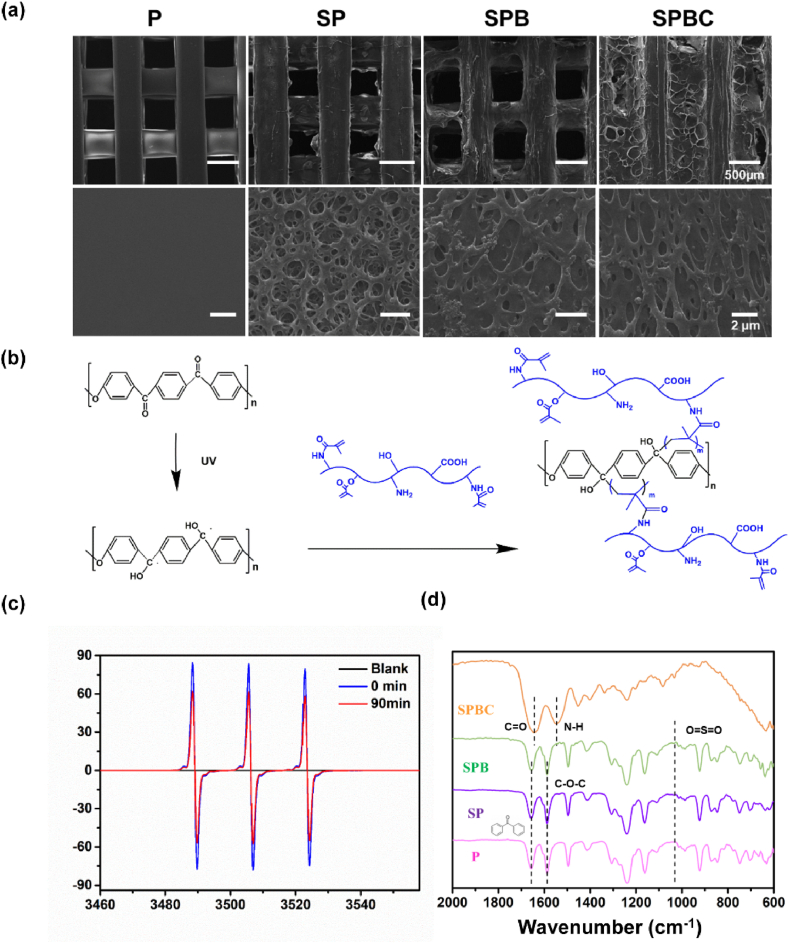
(a) SEM image of scaffolds. (b) Schematic illustration of the free radical generation and the surface modification of PEKK under UV irradiation. (c) EPR spectra of PEKK. (d) FTIR spectra of four scaffolds.

**Fig. 3:**
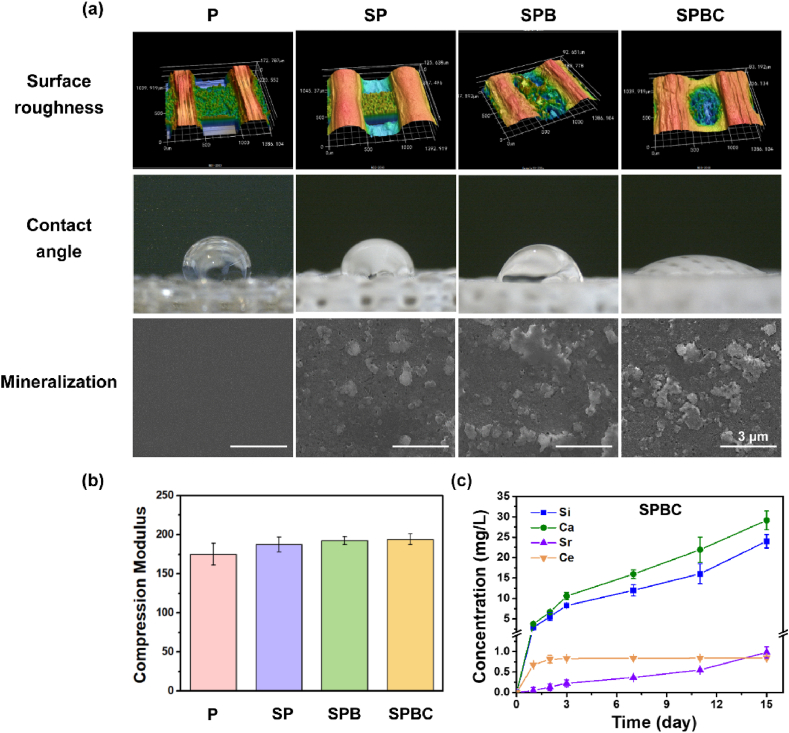
(a) Laser microscope 3D images of scaffolds, water contact angle of scaffolds and SEM after 14 days of mineralization. (b) Compression modulus of scaffolds. (c) Cumulative release of ions from SPBC.

SEM images of P, SP, SPB, and SPBC (Fig. 2a) revealed a macro-porous connectivity structure for all scaffolds. P exhibited a smooth surface with a filament width of 409.63 ± 40.12 μm and a pore size of 507.23 ± 34.63 μm. In contrast, SP and SPB scaffolds displayed rough surfaces with similar filament widths and pore sizes. Specifically, SP had a width of 451.27 ± 26.13 μm and a pore size of 403.82 ± 53.85 μm, while SPB had a width of 519.31 ± 40.45 μm and a pore size of 401.50 ± 35.92 μm. The surface of SPBC appeared rough, and the GelMA cryogel formed a uniform and highly interconnected network with numerous observable pores (Fig. S2b). The porosity of SPBC was measured as 54.21 %. The Sa of the scaffolds measured 0.36 ± 0.06 μm, 0.49 ± 0.05 μm, 1.73 ± 0.15 μm, and 2.22 ± 0.07 μm, respectively (Fig. 3a). Moreover, it was observed that the cryogels were tightly adhered to the inner wall of the pores, which may be attributed to the formation of covalent bonds between PEKK and GelMA (Fig. 2b). EPR spectra (Fig. 2c) indicated a 26 % decrease in the peak value of PEKK after 90 mins of UV illumination, confirming the generation of active radicals. FTIR spectra (Fig. 2d) confirmed characteristic peaks associated with PEKK in all four scaffolds. Specifically, the peaks at 1650 cm^−1^, 1490 cm^−1^, and 920 cm^−1^ corresponded to dibenzone moieties, while the peak at 1586 cm^−1^ corresponded to the stretching vibration of the C=C bond in the aryl ring. SP, SPB, and SPBC showed S-O characteristic peaks, with SPBC also exhibiting cryogel characteristic peaks. EDS mapping (Fig. S1b) revealed uniformly dispersed C (white) and O (red) elements on the surface of each scaffold. Additionally, SP, SPB, and SPBC scaffolds contained S (yellow), while SPB and SPBC also contained Si (green), Ca (pink), Sr (white), and Ce (purple).

The compressive moduli of the scaffolds (Fig. 3b) were measured as 175.14 ± 14.04 MPa, 187.66 ± 9.42 MPa, 192.41 ± 4.94 MPa, and 194.16 ± 6.85 MPa, respectively, which were similar to those of the cancellous bone (0.1–4.5 GPa) [25]. The water contact angles of the P, SP, SPB, and SPBC scaffolds (Fig. 3a) were determined as 107.59 ± 3.35°, 76.40 ± 1.33°, 71.44 ± 1.06°, and 25.54 ± 1.30°, respectively. ICP-OES analysis indicated that SPBC exhibited continuous release of Si, Ca, Sr, and Ce ions over a 15-day period (Fig. 3c). The cumulative release of Si and Ca ions was observed to be higher compared to that of Sr and Ce ions. Following a 14-day immersion period in SBF solution, the scaffolds exhibited the formation of apatite on their surfaces, as depicted in Fig. 3a. P did not exhibit any spherical apatite crystals and SP exhibited a limited presence. In contrast, SPB and SPBC revealed a notable abundance of uniformly distributed apatite crystals, providing further evidence for the hypothesis of bonding with natural bone.

### 3.2 Protein adsorption on scaffolds *in vitro* and *in vivo*

**Fig. 4:**
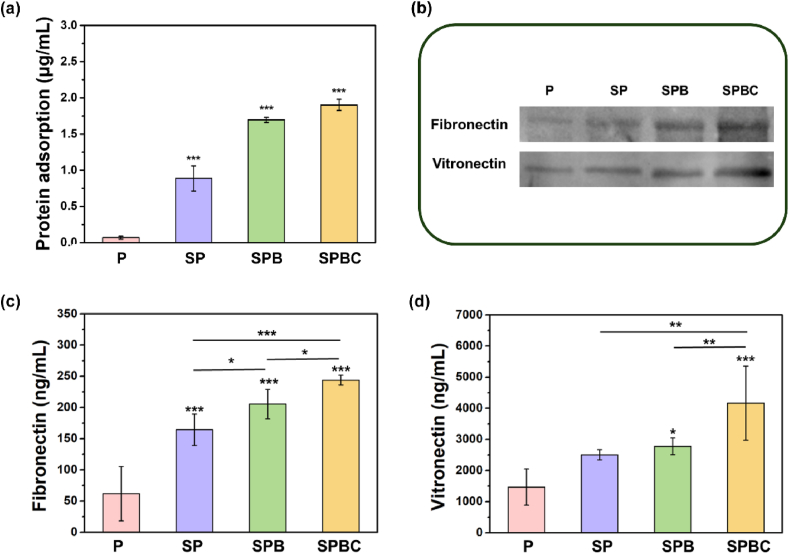
(a) Protein adsorption of different scaffolds *in vitro*. (b) The expression of fibronectin and vitronectin *in vivo* by Western blot assay and the concentration of (c) fibronectin and (d) vitronectin by ELISA. n = 3, ∗: P < 0.05; ∗∗: P < 0.01; ∗∗∗: P < 0.001.

The adsorption of proteins on the surface of the material can directly mediate cell-substrate interactions [26]. *In vitro* protein adsorption assays (Fig. 4a) demonstrated that the concentrations of adsorbed BSA on the surface of P, SP, SPB, and SPBC were tested to be 0.07 ± 0.02 μg/mL, 0.89 ± 0.17 μg/mL, 1.70 ± 0.03 μg/mL, and 1.90 ± 0.08 μg/mL, respectively. As for *in vivo* experience, four scaffolds were implanted into the rat femoral defects for 24 h. Western blotting results (Fig. 4b) indicated that the adsorption of fibronectin and vitronectin by the P, SP, SPB and SPBC increased sequentially. The quantitative results obtained *via* ELISA (Fig. 4c and d) showed that the adsorption concentrations of fibronectin for P, SP, SPB, and SPBC were 61.85 ± 43.65 ng/mL, 164.36 ± 24.96 ng/mL, 205.33 ± 23.62 ng/mL and 243.82 ± 7.78 ng/mL, while those of vitronectin were 1468.02 ± 582.65 ng/mL, 2505.85 ± 162.00 ng/mL, 2779.53 ± 265.93 ng/mL, and 4166.53 ± 1189.54 ng/mL, respectively.

### 3.3 Adhesion, proliferation and differentiation of rMSCs on scaffolds

**Fig. 5:**
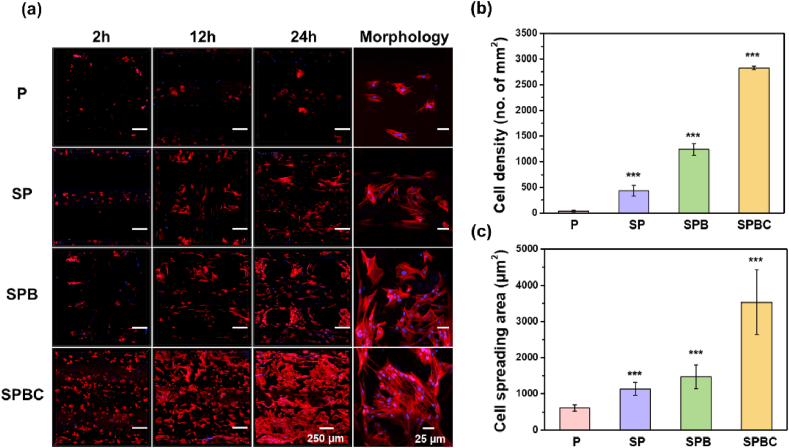
(a) Representative images of actin filaments of rMSCs cultured on the surface of scaffolds for 2 h, 12 h and 24 h. (b) Cell density and (c) cell spreading area of the adhered rMSCs.

The attachment and growth of rMSCs on different scaffolds was detected by cytoskeletal staining (Fig. 5). It was observed that following a culture of rMSCs on the scaffold surface for 2 h, 12 h and 24 h, their number showed a time-dependent increase. At 24 h, the cell density on the scaffold surfaces followed the order of SPBC > SPB > SP > P. Moreover, the spreading area of cells on the scaffold surfaces exhibited similar changes. Cells exhibited different morphologies after seeded on different scaffolds. At 2 h, the cells on the surface of P, SP and SPB showed an unstretched spherical shape, whereas a small number of cells on the surface of SPBC started to spread and the total number of cells became more. At 12 h, cell morphology further stretched out, and by 24 h, the number of cells on the surface of P was low, exhibiting spherical morphologies that had not been stretched. In contrast, cells on the SP and SPB surfaces were polygonal. Notably, cells on the surface of SPBC all extended pseudopods that were spindle-shaped and spread out over the scaffold.

**Fig. 6:**
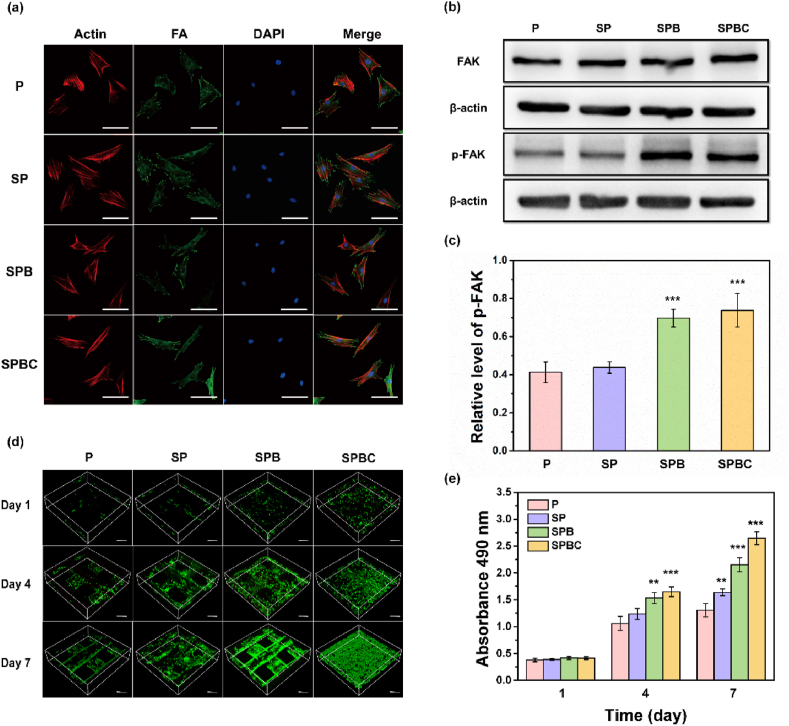
(a) Immunofluorescent staining characterization of rMSCs with cytoskeleton (red), focal adhesion (FA, green) and nuclei (blue). Scale bar = 100 μm. (b) Western blotting analysis of FAK and phospho-FAK (Tyr397, Tyr925) in rMSCs. (c) Semi-quantitative expression of p-FAK. (d) Live/dead staining and (e) cell viability of rMSCs. Scale bar = 450 μm. (∗∗∗p < 0.001). (For interpretation of the references to color in this figure legend, the reader is referred to the Web version of this article.)

The distribution of focal adhesions (FA) in cells co-cultured with scaffolds for 24 h was detected by immunofluorescence staining. As shown in Fig. 6a, the FA of P and SP were clustered into dots, and the area of a single spot was small. Conversely, the area of FA increased after stimulation by SPB and SPBC, indicating higher maturity. Western blotting analysis (Fig. 6b) showed that FAK was expressed in all cells during the spreading process, and the protein expression level of FAK phosphorylation at Y397 (p-FAK) on SPB and SPBC was significantly up-regulated. Semi-quantitative analysis (Fig. 6c) showed that the expression level of p-FAK in the cells co-cultured with SPB and SPBC was 1.7 and 1.8 times that of P, respectively.

CCK-8 assay and live-dead staining were employed to assess the proliferation of rMSCs on the scaffolds (Fig. 6d and e). The proliferation rate of cells cultured on all four scaffolds exhibited an increase over time. Specifically, the proliferation rate on SPB and SPBC was higher than that on P on day 4 and the rate on SP, SPB and SPBC was higher than that on P on day 7.

**Fig. 7:**
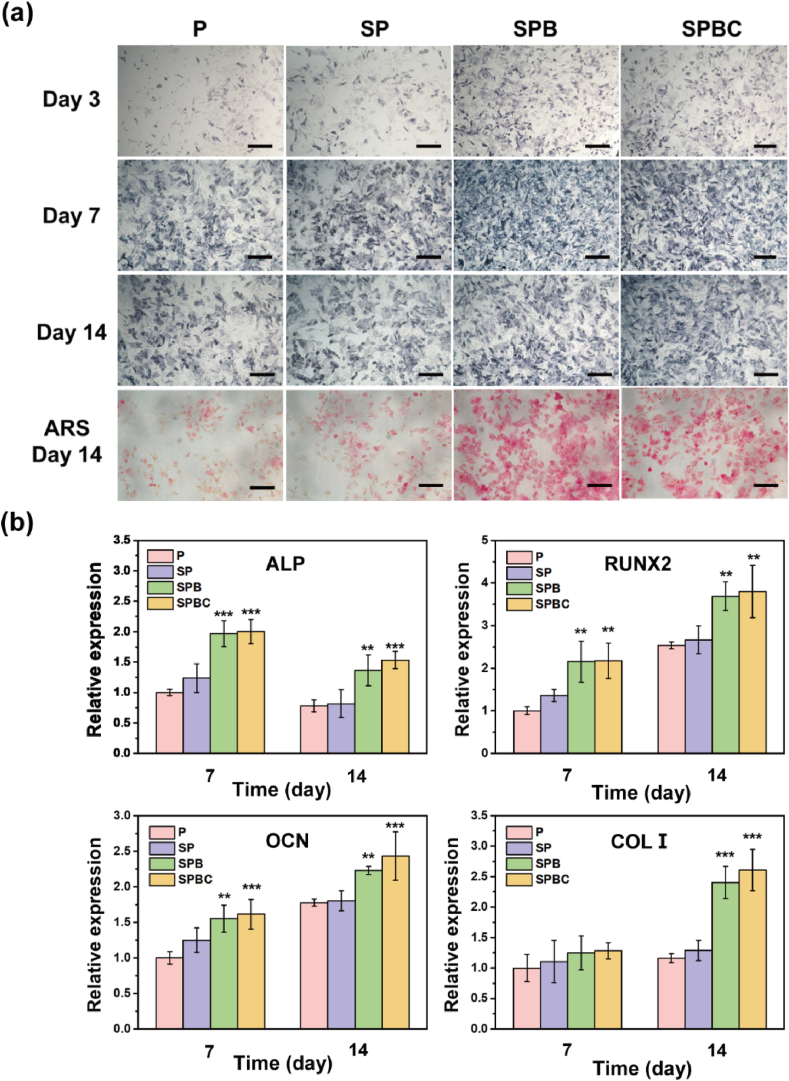
(a) ALP and ARS staining images. Scale bar = 200 μm. (b) Osteogenic-related gene (ALP, Runx2, Col I, and OCN) analysis of rMSCs after incubation for 7 and 14 days.

The osteogenic activities of various scaffolds were assessed primarily through ALP, ARS, and qPCR (Fig. 7, S3a and S3b). As demonstrated by the ALP staining on days 3, 7, and 14, the blue-purple color was darker in the SPB and SPBC groups compared to P and SP. Furthermore, the intracellular ALP activity and calcium deposition were significantly higher in the SPB and SPBC groups compared to P and SP. The expression levels of key osteogenic genes, including Runx2, ALP, Col I, and OCN, were measured in the cells of the scaffold using qPCR. The results demonstrated a time-dependent increase in the expression of Runx2, Col I, and OCN genes, while the ALP gene exhibited a decrease at 14 days. Importantly, compared to P, the SPB and SPBC scaffolds exhibited significant upregulation of ALP, Runx2, Col I, and OCN gene expression at each time point with different increments, which convincingly demonstrated the ability of SPB and SPBC scaffolds to promote osteogenic differentiation.

### 3.4 Residence, proliferation and polarization of macrophages on scaffolds

**Fig. 8:**
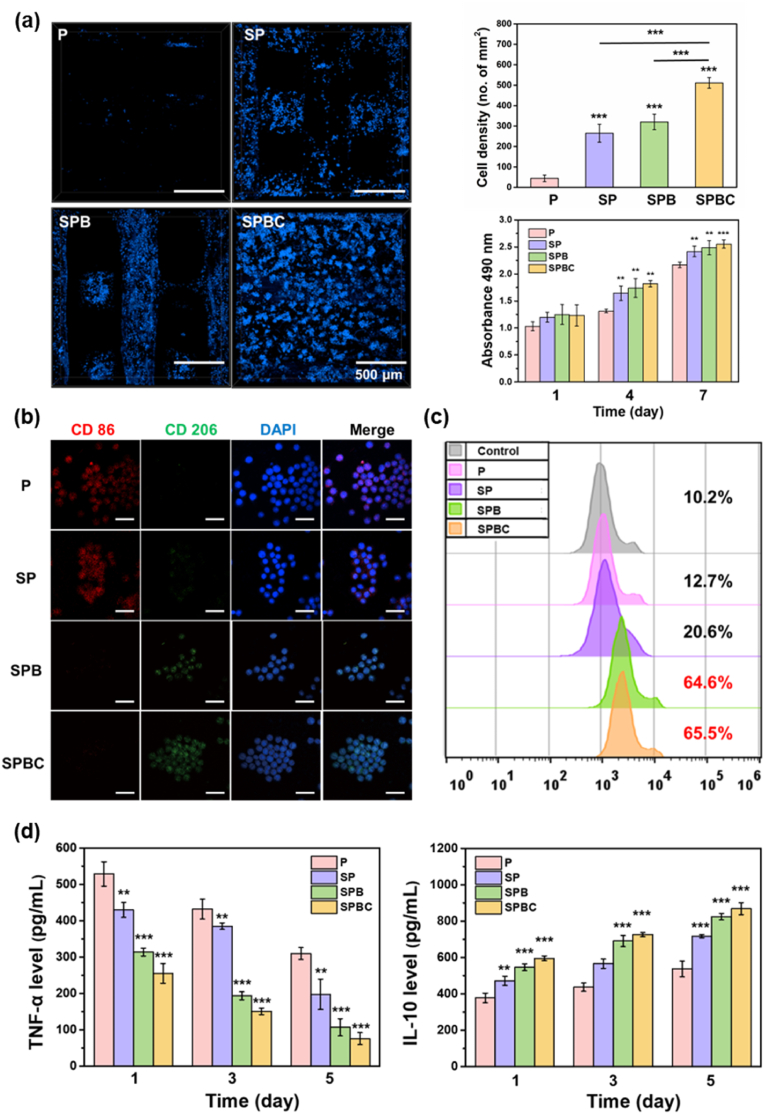
(a) The distribution, cell number and proliferation of RAW264.7 cells on the scaffolds. (b) Representative CLSM images of RAW264.7 cells cultured on different scaffolds for 3 days (Red: CD86; Green: CD206; Blue: cell nuclei, Scale bar = 30 μm); (c) Percentages of CD206-positive RAW264.7 cells by flow cytometry; (d) TNF-α, and IL-10 secreted by RAW264.7 cells. (For interpretation of the references to color in this figure legend, the reader is referred to the Web version of this article.)

Fig. 8a showed the distribution and the number of cells after macrophages cultured on the scaffold for 3 days. When the same concentration of cell suspension was added to the scaffold, the number of cells was lowest on P, highest on SPBC and similar on SP and SPB. CCK-8 was employed to assess the proliferation of macrophages following various culture durations. The proliferation rate of cells cultured on all four scaffolds exhibited a time-dependent increase.

To further investigate the polarization of macrophages on scaffolds, cells were seeded onto the scaffolds and cultured for 3 days (Fig. 8b and c). Immunofluorescence staining results revealed that the number of CD86-positive cells was highest on P, followed by SP. Conversely, the number of CD206-positive cells was higher on SPB and SPBC. Flow cytometry analysis demonstrated that the percentage of M2 macrophages on the four scaffolds was measured as 12.7 %, 20.6 %, 64.6 %, and 65.5 %, respectively. With the prolongation of culture time, the secretion of the M1 marker, tumor necrosis factor-alpha (TNF-α), gradually decreased, while the secretion of the M2 marker, interleukin-4 (IL-4), exhibited a gradual increase (Fig. 8d). Notably, the secretion of TNF-α by macrophages followed the order of P > SP > SPB > SPBC at all time intervals and the secretion of IL-10 displayed an opposite trend.

### 3.5 Scaffolds for bone defect repair *in vivo*

**Fig. 9:**
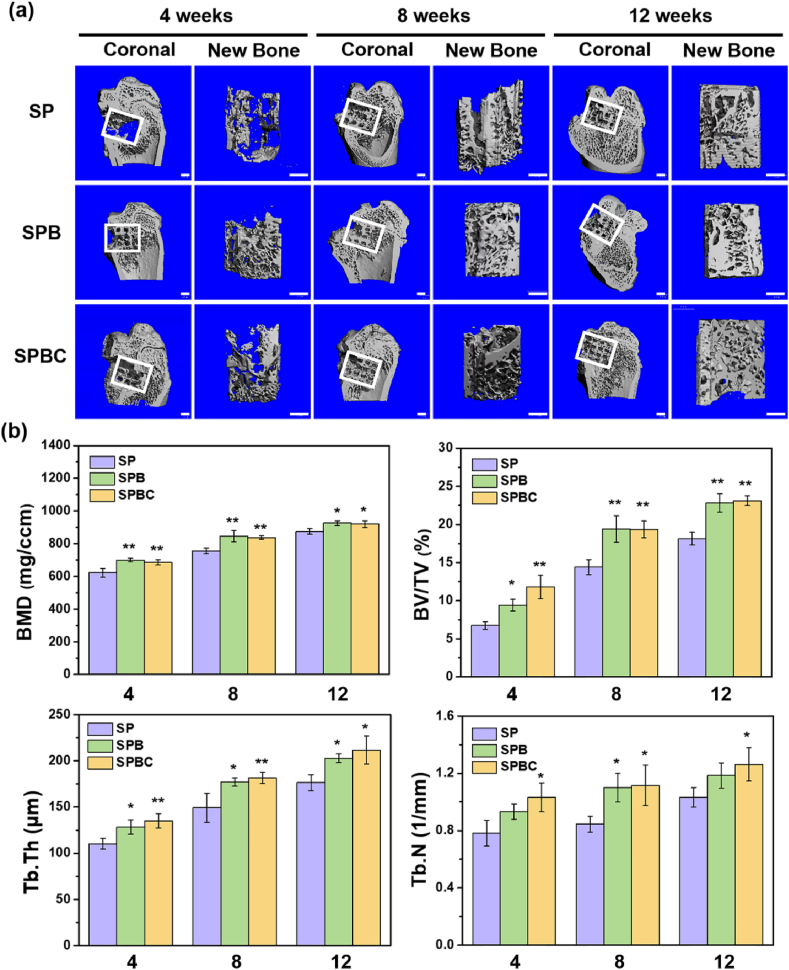
(a) 3D micro-CT coronal views of femur defect area and reconstruction regenerated bone around defects. Scale bar = 1 mm. (b) Quantitative analysis of the bone volume (BMD), bone volume fraction (BV/TV), bone trabecular thickness (Tb.Th) and trabecular number (Tb.N).

Based on the data above, SP, SPB, and SPBC were selected for experiments on distal femoral defects in rats. Due to its radiopacity, the scaffolds were not visible at the implantation site. Micro-CT images (Fig. 9a) revealed that at 4 weeks, only a small amount of irregular new bone tissue existed at the defect of SP, whereas new bone in the shape of a scaffold had already appeared at the implantation site of SPB and SPBC, growing from the periphery towards the center. At 8 weeks, there was a trend of new bone growth into the implantation sites of the scaffold groups, although no new bone appeared at the central region. At 12 weeks, grid-shaped new bone tissue, observed at the implantation site of SP, was further growing towards the center, but there was still a defect. The new bone tissue at SPB further extended with a reduced vacancy. Notably, the SPBC implantation site was essentially covered.

Quantitative analysis by Micro-CT (Fig. 9b) demonstrated that BMD, BV/TV, Tb.N and Tb.Th of new bone tissue on all four scaffolds increased over time. Specifically, the BV/TV of SPBC after 4 weeks of implantation was 12.48 % ± 0.38 %, which was 1.8 times higher compared to SP (6.95 % ± 0.95 %), and the BV/TV of SPB was 9.42 % ± 0.78 %, representing a 1.4-fold increase over SP. At 12 weeks, the BV/TV for SP, SPB, and SPBC were determined to be 18.30 % ± 1.59 %, 22.14 % ± 2.59 %, and 23.11 % ± 0.65 %, respectively. Both SPB and SPBC exhibited significantly higher BV/TV compared to SP, although the difference between SPB and SPBC was not statistically significant. At 4 weeks after implantation, there was no notable difference in BMD among the three groups, but the BMD of SPB (0.93 ± 0.02 g/ccm) and SPBC (0.92 ± 0.02 g/ccm) were higher than that of P (0.85 ± 0.03 g/ccm) at 12 weeks. Regarding to the bone trabecular thickness (Tb. Th) and number (Tb. N), SPB and SPBC were higher than P at predetermined time points.

**Fig. 10:**
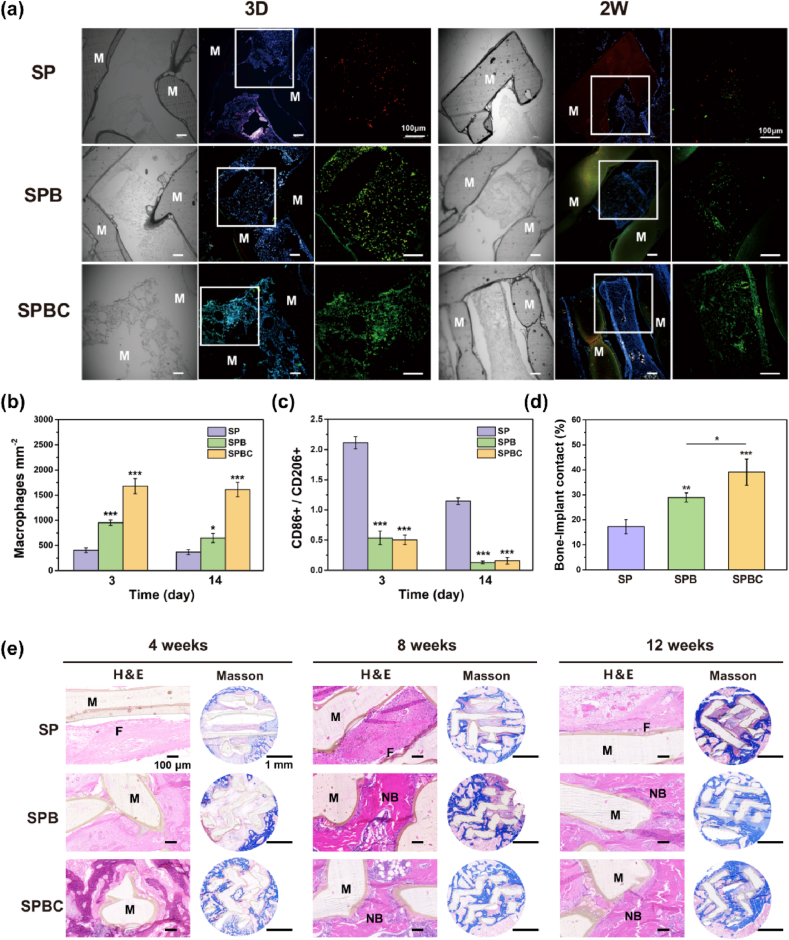
(a) Representative immunofluorescence images of DAPI (blue), CD86 (red) and CD206 (green) stained at 3 days and 2 weeks postoperatively. (b) Quantitative analysis of macrophages (CD68 and CD206). (c) The proportion of CD206 and CD86 at 3 days and 2 weeks. (d) bone-implant contact at 12 weeks in Masson's Trichrome staining. (e) H&E and Masson's Trichrome staining images. Key: M, Materials; NB, new bone; F, fibrous capsule. (For interpretation of the references to color in this figure legend, the reader is referred to the Web version of this article.)

After 3 days of *in vivo* implantation, macrophages were observed surrounding the scaffolds in all three groups (Fig. 10a–c). Notably, a high number of macrophages adhered to the cryogel of SPBC, while fewer macrophages were observed inside the SP and SPB. The presence of cryogel was still noticeable in the SPBC group after 2 weeks (Fig. S3c). Furthermore, the ratio of M1/M2 macrophages was 0.13 in SPB and 0.15 in SPBC, which was higher than that in SP (1.15), with no significant difference between SPB and SPBC.

To further evaluate the therapeutic effects of scaffolds, histological analysis was performed as Fig. 10e. H&E staining revealed a large amount of fibrous tissue and a small amount of new bone around the SP after 4 weeks. The thickness of the fibrous tissue gradually decreased with the time of implantation, but it was still observed at 12 weeks. In contrast, no significant inflammatory cells were detected at the edges of SPB and SPBC scaffolds. Masson staining demonstrated that after 4 weeks of implantation, little new bone was found around SP and there was a noticeable gap between the tissue and the scaffold. SPB scaffolds exhibited more new bone formation, although a gap still remained. While SPBC scaffolds already exhibited new bone growth within the scaffold with no observable gap between the newly formed bone and the implant. After 8 weeks of implantation, all groups showed increased amounts of new bone. At 12 weeks, the new bone in the SPBC penetrated the entire defect area, grew from the periphery into the center of the scaffold, and formed synostosis (Fig. 10e). Additionally, a magnified view (Fig. S3d) revealed that clear gaps were obvious between the scaffolds and collagen fibers in the SP and SPB groups, while the SPBC group exhibited a tight interface with no gaps between the collagen fibers and the implant. The bone-to-implant contacts were also calculated to be 17.27 %, 28.98 % and 39.12 %, respectively (Fig. 10d).

## 4 Discussion

Previous studies have established PEKK as a commonly used material for orthopedic implants. However, its inherent biological inertness often leads to poor osseointegration, resulting in implant surgery failures [27]. The combination of BG with poly(ether-ether-ketone) through laser powder bed fusion, electrophoretic deposition, and dip-coat has been reported [[28], [29], [30]]. In this study, a porous PEKK composite scaffold with enhanced biological activity was developed using fused deposition modeling. It is reported that as the temperature rises, the thermal decomposition process of PEKK primarily initiates with the random chain breaking of ether and ketone bonds along the molecular chain, forming benzene, ether or carbonyl radicals [31]. This is followed by the crosslinking of benzene-ring-containing free radicals, leading to the formation of a structure similar to graphite with good stability. From the TG it can be seen that the residual rate of PEKK is 59.0 % and that of PEKK/BG is 62.6 % at 800 °C. The observed difference between these values may be attributed to the incorporation of BG with high temperature resistance. Based on the above analysis, the SPB scaffold contained 3.6 wt% BG (Fig. S2a). When PEKK is immersed in concentrated sulfuric acid, the sulfonation reaction occurs. The intermolecular forces will decrease after the introduction of -SO₃H into the PEKK molecular chain. Due to the inhomogeneity, the region with higher degree of sulfonation will trigger phase separation from the region with lower degree, so many micropores appear at the interface between different phases [32,33]. In addition, concentrated sulfuric acid can penetrate into the molecular chain of PEKK and expand the molecular chain spacing, leading to the swelling of PEKK. During this process, the stress inside the material is distributed unevenly, creating tiny cracks and pores in areas of concentration [34]. Hence, the surface of the sulfonated scaffold had a porous structure, which significantly improved the surface roughness of the scaffold (Fig. 3a). Fig. 2b represented the process of free radical generation of PEKK under UV irradiation and subsequent binding to GelMA. Specifically, the main chain of PEKK contains diphenyl ketone, with a structure similar to that of a photoinitiator [11]. After 90 min of UV illumination, PEKK could generate active radicals to graft GelMA onto the PEKK surface without the additional photo initiators (Fig. 2c and d). As for the cryogel filled in the pores of scaffolds, they were crosslinked in a freezing environment to form an interconnected macroporous structure for subsequent cell residence. Moreover, SPBC facilitated a continuous release of Si ion (24.02 mg/L), Ca ion (29.14 mg/L), Sr ion (0.98 mg/L) and Ce ion (0.84 mg/L) from the scaffold. It is reported that Si ion can increase metabolic activity within a range of 15–50 ppm [23,35]; Ca ion (≤4 mM) can stimulate ALP expression and proliferation of primary osteoblasts [36]; Sr ion (5–500 μM) can promote osteogenic differentiation of hASCs by promoting ALP activity, extracellular calcium deposition, and expression of osteogenesis-related genes [37]; The addition of cerium oxide nanoparticles to 70S bioglass scaffolds can enhance osteoblastic differentiation and collagen production of rMSCs, and significantly reduced the expression of pro-inflammatory genes L-1β, IL-6 and TNF-α [38]. Based on the above research, the loading and release ions of SPBC were within the appropriate concentration. In addition, it exhibited excellent mineralization *in vitro*, which have potential to improve the bioactive of PEKK (Fig. 3a). The compression modulus of four scaffolds (Fig. 3b) were similar to that of cancellous bone, which reduced the stress shielding effect of bone tissue on scaffolds.

**Scheme 1:**
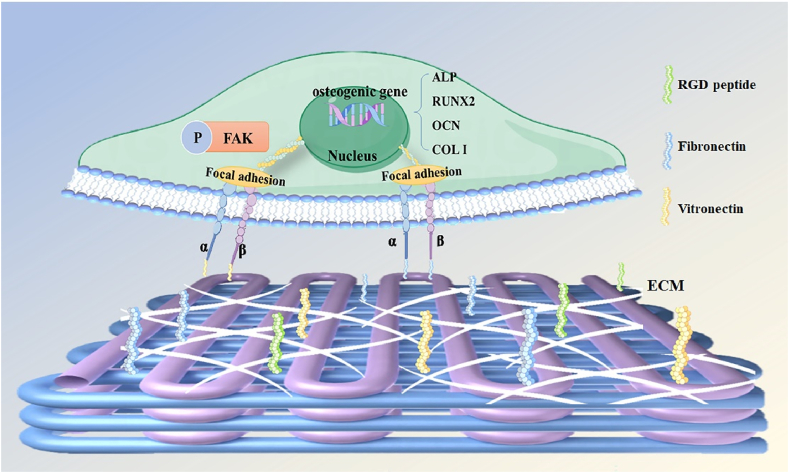
Schematic diagram of SPBC regulated the cell behavior of rMSCs on the surface.

Proteins adsorption is the initial event after implantation, and cells primarily bind to the proteins adsorbed on the scaffold surface through integrins on the cell membrane, rather than direct contacting the material [39,40]. Therefore, increasing the amount of protein adsorbed to the surface of scaffold is important for improving cell adhesion. Due to its smooth surface, PEKK exhibited a weak protein adsorption capacity *in vitro* (Fig. 4a). In contrast, SP showed slight improvement due to the sulfonation reaction, which enhanced the surface roughness and hydrophilicity of the scaffold (Fig. 3a). SPBC filled the pores with cryogel, resulting in a significant increase in the contact area between the scaffold surface and the surrounding solution. Additionally, the GelMA cryogel enhanced the interaction between the scaffolds and proteins, directly improving the protein adsorption of SPBC. Following implantation *in vivo*, SPBC was found to adsorb the highest levels of fibronectin and vitronectin compared to the other three groups (Fig. 4b–d). Both of the two proteins are representative ECM proteins, which have integrin recognition sites [41,42]. rMSCs can bind to fibronectin through integrin α5β1 and vitronectin through αvβ3 [43,44], thereby adhering to the surface of SPBC. The adhesion of rMSCs plays a crucial role in the early stages of osseointegration [45]. After 24 h of co-culture with rMSCs, cells cultured on P/SP scaffold became flattened and formed more scattered adhesion plaques, while on SPB/SPBC, their F-actin filaments traversed the cell body and formed bundles at the branching points, resulting in spindle-shaped cells (Fig. 5a–c). Since Focal Adhesion Kinase (FAK) is associated with integrin receptors and regulates cellular activities [46], the expression of phosphorylated FAK (p-FAK) was detected (Fig. 6b–c). The cells co-cultured with SPB and SPBC significantly up-regulated the expression during the spreading process, indicating the incorporation of BG played a crucial regulatory role in cell adhesion. It has been reported that integrins require binding of divalent cations to their ligands to change conformation, allowing ECM binding [47]. The wire-mixed PEKK with BG by melt drawing could sustainably release divalent ions such as Ca^2+^ and Sr^2+^ (Fig. 3c), thereby promoting cell adhesion to the protein layer on the scaffold. Furthermore, SPB/SPBC elevated the phosphorylation level of FAK at the Y397 site, regulated the maturation of cytoskeleton and adhesion plaques and thus facilitated cell spreading (Scheme 1).

The regulation of the implant on macrophages significantly influences the outcome of osseointegration [48]. Macrophages on the surface of P tended to maintain a pro-inflammatory M1 phenotype, leading to the fusion of macrophage into multinucleated giant cells, increased release of fibrosis-enhancing cytokines, and subsequent promotion of fiber encapsulation. This prevented the implant from contacting with bone tissue, and seriously impeded bone regeneration. In comparison to P, the micro/nano structure of SP's surface increased hydrophilicity and roughness, exerting an inhibitory effect on M1 macrophage [49]. SPB and SPBC obviously promoted the polarization of macrophages towards the M2 phenotype at the initial stage (3 days), inhibited the production of pro-inflammatory factors (TNF-α) and promoted the secretion of anti-inflammatory factors (IL-10) (Fig. 8b–d). It might be bioactive ions released from the scaffold play a key role. SiO_3_^2−^, Ca^2+^, and Sr^2+^ released from BG have been demonstrated to suppress the activated inflammatory MAPK and NF-κB signaling pathway [50]. In the physiological environment, Ce^3+^ can also switch the oxidative state between Ce^4+^ and Ce^3+^, eliminate free radicals, and regulate the oxidative state to induce anti-inflammation [51]. Under the combined action of various bioactive ions, macrophages would be promoted to M2 polarization, and released more cytokines that promoted osteogenic differentiation of rMSCs, which was conducive to bone regeneration.

Moreover, the osteogenic differentiation of rMSCs adhering to the scaffold is an important factor, which directly affects the new bone regeneration. The expression of Runx2, ALP, Col I and OCN genes was examined after 14 days (Fig. 7b). ALP is an early marker of osteoblast differentiation and functional maturation due to its ability to hydrolyze pyrophosphate into phosphate, which in turn promotes calcium deposition [52]. During a 14-day co-culture with the scaffold, ALP activity and gene expression initially increased and late decreased, signifying that the scaffold significantly advanced the timing of cell differentiation and maturation. Runx2, Col I and OCN, related to matrix mineralization, are commonly utilized as markers of late-stage osteogenesis. Specifically, Runx2 is a specific transcription factor that regulates the expression of matrix proteins in osteoblasts. Col I is a major component of bone matrix that provides sufficient mineralization sites, and OCN is a specific non-collagenous bone matrix protein, which can maintain the normal bone mineralization rate [[53], [54], [55]]. After co-culture of rMSCs with scaffolds for 14 days, the gene expression of Runx2, Col I and OCN gradually increased with time. Compared with P, the changes in surface morphology of SP favored the differentiation of rMSCs into the osteogenic lineage, although there was no significant difference. The expressions of osteogenesis-related genes on SPB and SPBC were significantly higher than that on P/SP. Alizarin red staining showed that after co-culture with SPB/SPBC, calcium salt was deposited on the surface of rMSCs, leading to an increase in calcium nodules (Fig. 7a and S3b). This might be the fact that Ca^2+^ released from SPB/SPBC scaffolds can activate the intracellular MAPK signaling pathway by contacting the extracellular G-protein coupled receptor calcium sensing receptor (CaSR), which promotes the proliferation and differentiation of rMSCs and increases the mineralization and deposition of extracellular matrix [56]. In addition, Sr^2+^ stimulates the proliferation of rMSCs through Wnt/β-catenin signaling pathway, and Ce generally promotes osteogenesis by activating BMP-TGFβ signaling pathway or SMAD1/5/8 signaling pathway [[57], [58], [59]]. The addition of BG significantly enhanced the capacity of PEKK-based scaffolds to stimulate bone differentiation.

*In vivo* experiments were carried out to reconfirm the immune response and bone regeneration of the scaffolds. More macrophages were found within SPBC due to the cryogel filling. Regarding the inflammatory response (Fig. 10), SPBC exhibited a higher percentage of M2-type macrophages than the other two groups at 3 days post-operation, indicating the anti-inflammatory properties of the scaffold. After 2 weeks, M2 macrophages still remained the dominant phenotype around SPBC. 4 weeks later, H&E staining revealed only a small amount of fibrous tissue formed around the SPBC, suggesting a prolonged anti-inflammatory effect of the SPBC. The proportion of M1 macrophages gradually decreased in the SP based on the organism's natural repair process, but a thicker fibrous capsule appeared at 4 weeks, indicating that the undesirable inflammatory reaction occurred in SP. The anti-inflammatory property of SPB was intermediate between SP and SPBC, which may be attributed to the addition of BG and the significant influence of adsorbed proteins on the inflammatory response of the samples. It has been reported that adsorbed FN-mediated cell adhesion and its conformational changes promoted the anti-inflammatory response [60]. Subsequent osteogenic evaluation revealed that new bone growth originated from the periphery and extended to the interior of the scaffolds, facilitated by the pore connectivity and macroporous structure, which demonstrated the favorable osteoconductivity of all three scaffolds (Fig. 9) [61]. BMD, BV/TV, Tb. N and Tb. Th were significantly higher in SPB and SPBC than in SP at 4, 8, and 12 w after implantation, indicating that bioactive ions released from SPB and SPBC accelerated new bone regeneration and enhanced osteoinductivity. Remarkably, following a 12-week implantation, SPBC exhibited excellent osseointegration and substantial bone repair in the femoral defect.

## 5 Conclusion

In this study, a novel PEKK-based scaffold (SPBC) was developed through mixing PEKK with BG, sulfonating by sulfuric acid and loading cryogel into pores. SPBC demonstrated superior fibronectin and vitronectin adsorption, enhancing cell adhesion. Additionally, SPBC exhibited an increased phosphorylation of FAK at the Y397 site, which accelerated cell spreading and ultimately improved the osteointegration effect of PEKK. Furthermore, the incorporation of BG into scaffolds facilitated the sustained release of bioactive ions, enhancing the polarization of M2 macrophages and the secretion of anti-inflammatory cytokines, as well as promoting the osteogenic differentiation of rMSCs. Animal experiments further confirmed the significant promotion of bone formation and osteointegration by SPBC. In summary, the PEKK-based scaffold exhibited excellent cytocompatibility, promotion of cell adhesion, anti-inflammatory and osteogenesis properties, highlighting its significant potential in the field of bone repair.

## CRediT authorship contribution statement

**Qianwen Yang:** Writing – review & editing, Writing – original draft, Project administration, Methodology, Investigation. **Anbei Chen:** Methodology, Investigation, Data curation. **Xin Zhang:** Investigation. **Zhaoying Wu:** Supervision, Funding acquisition, Conceptualization. **Chao Zhang:** Supervision, Resources, Funding acquisition.

## Declaration of competing interest

The authors declare that they have no known competing financial interests or personal relationships that could have appeared to influence the work reported in this paper.

## Data Availability

Data will be made available on request.
