# Supplementary material for: Functional poly(ether-ketone-ketone) composite scaffold with enhanced cell-material interaction, anti-inflammatory and osteogenesis for facilitating osteointegration and bone regeneration
Source: Mater Today Bio. 2025 Jan 29;31:101533. doi: 10.1016/j.mtbio.2025.101533 (PMC11835654; doi:10.1016/j.mtbio.2025.101533)
Supplement: Multimedia component 1 [file mmc1.docx]

**Supporting Information**

**Functional poly(ether-ketone-ketone) composite scaffold with enhanced cell-material interaction, anti-inflammatory and osteogenesis for facilitating osteointegration and bone regeneration**

*Qianwen Yang^1^, Anbei Chen^1^, Xin Zhang^1^, Zhaoying Wu^1, *^ and Chao Zhang^1, *^*

^1^ School of Biomedical Engineering, Shenzhen Campus, Sun Yat-Sen University, Shenzhen, Guangdong, 518107, China

* Correspondence should be addressed to:

E-mail: wuzhy37@mail.sysu.edu.cn; zhchao9@mail.sysu.edu.cn

**Table S1 List of primers for target genes**

| **Genes** | **Primer sequences (5' to 3')** |
| --- | --- |
| **GAPDH-S** | AAATGGTGAAGGTCGGTGTGAAC |
| **GAPDH-A** | CAACAATCTCCACTTTGCCACTG |
| **ALP-S** | AGCGACACGGACAAGAAGC |
| **ALP-A** | GGCAAAGACCGCCACATC |
| **RUNX2-S** | GCACCCAGCCCATAATAGA |
| **RUNX2-A** | TTGGAGCAAGGAGAACCC |
| **COL 1-S** | CCTGAGCCAGCAGATTGAGA |
| **COL 1-A** | TCCGCTCTTCCAGTCAGA |
| **OCN-S** | GACAGCAACGGGAAGACC |
| **OCN-A** | CAGGCTGGCTTTGGAACTC |


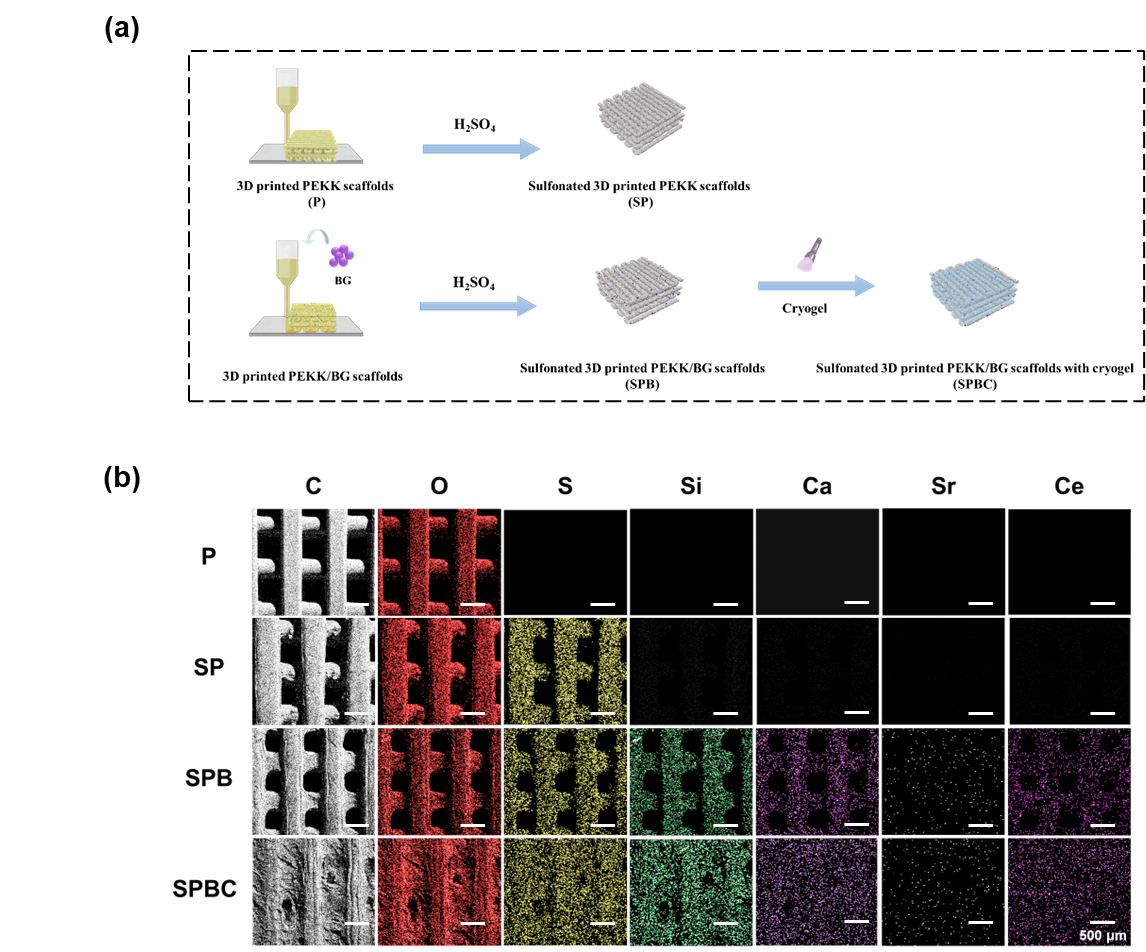


**Fig. S1** (a) The synthetic scheme of samples. (b) Element mapping of different scaffolds.


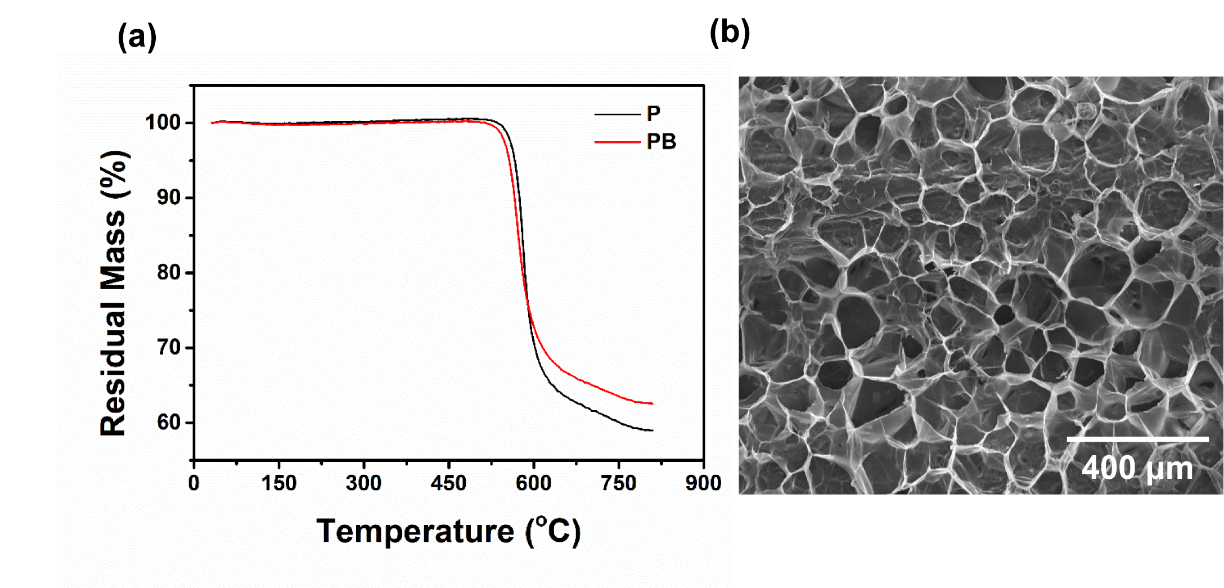


**Fig. S2** (a) TG curve of PEKK (P) and PEKK/BG (PB). (b) SEM image of cryogel in the SPBC.


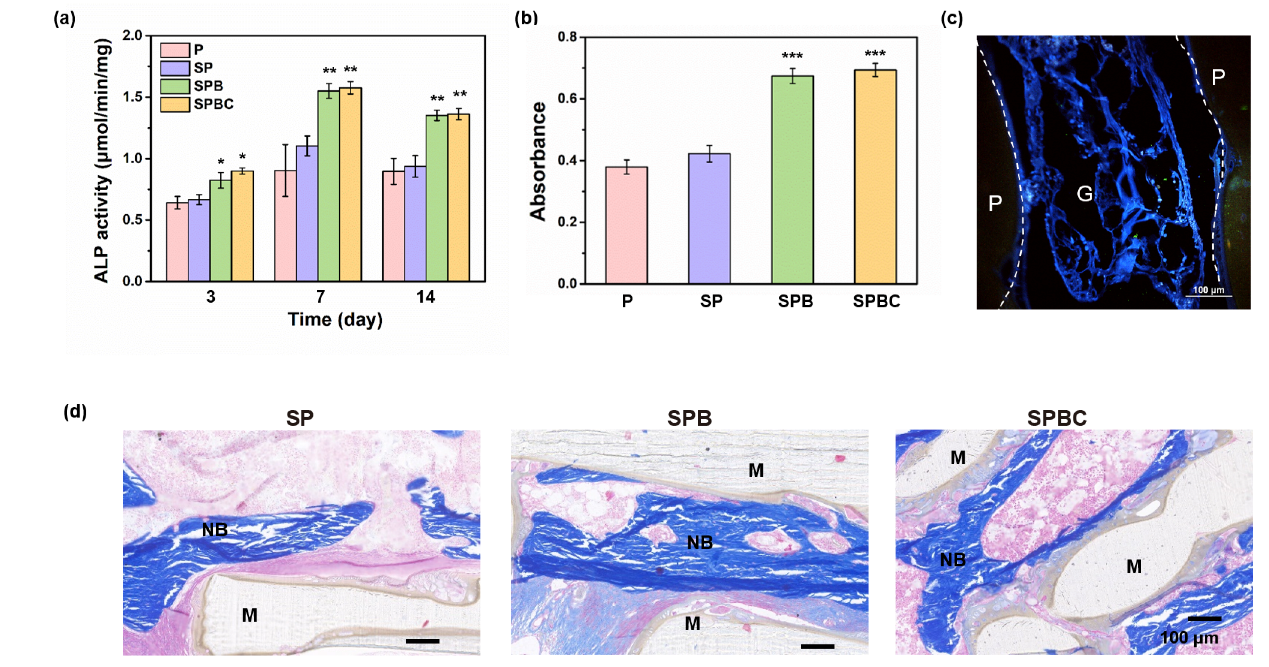


**Fig. S3** (a) ALP activity of rMSCs after 3-, 7- and 14-days induction on the different scaffolds. (b) Calcium deposition of rMSCs on different scaffolds after culture for 14 days. (c)  A confocal image of cryogel in the SPBC at 2 weeks. (d) Masson's Trichrome staining of scaffolds at 12 weeks. Key: P, PEKK; G, Cryogel; M, Materials; NB, new bone.
